# Host genetic background influences the severity of disease in *Schistosoma haematobium* infections

**DOI:** 10.1051/parasite/2026013

**Published:** 2026-03-23

**Authors:** Boris Sègnito A.E. Savassi, Samoussou-Dine K. Mahaman, Djèlili Biaou, Nélia Luviano Aparicio, Moudachirou Ibikounlé, Eve Toulza, David Courtin, Geoffroy Hounkanrin, Achille Massougbodji, Jérôme Boissier

**Affiliations:** 1 IHPE, Université de Perpignan Via Domitia, CNRS, IFREMER, Université de Montpellier Perpignan France; 2 Centre de Recherche pour la Lutte contre les Maladies Infectieuses Tropicales (CReMIT/TIDRC), Université d’Abomey-Calavi Bénin; 3 Institut de Recherche Clinique du Bénin (IRCB) Abomey-Calavi Bénin; 4 Institut de Recherche Pour le Développement (IRD), UMR Intertryp IRD-CIRAD-UM 34398 Montpellier Cedex 5 France; 5 Service de Radiologie et Imagerie Médicale, Centre National Hospitalier et Universitaire Hubert Koutoukou Maga (CNHU-HKM) Cotonou Bénin

**Keywords:** Urogenital schistosomiasis, Morbidity, Genetic factors, *TNF-α* promoter, Ultrasound, Benin

## Abstract

Host genetic factors influence the severity of infectious diseases, including schistosomiasis, which are major public-health burdens in Africa. While the role of host genetic background in *Schistosoma mansoni* infection has been clearly established, this link remains poorly explored for *S. haematobium* infections (*Sh*). Therefore, this study aims to investigate the relationship between genetic background and morbidity associated with urogenital schistosomiasis using a candidate gene approach. We analyzed urine samples from 334 Beninese men, measuring urinary eosinophil cationic protein (ECP) by ELISA as a marker for bladder inflammation. Abdominopelvic ultrasonography was performed in a subgroup of 146 participants (69 *Sh*-positive and 77 *Sh*-negative) to assess morbidities associated with *Schistosoma* infection. Blood samples were analyzed for TNF-α levels by ELISA and for TNF-α promoter polymorphisms by sequencing to assess associations between genetic variation and morbidity. Results showed that 25.4% of *Sh*+ had significantly higher mean *TNF-α* (U = 5888; *p* = 0.0098) and ECP (U = 912.5; *p* < 0.0001) levels than *Sh*−. Positive correlations were observed between egg count and both ECP (Tau = 0.4016; *p* < 0.0001) and *TNF-α* levels (Tau = 0.2238; *p* = 0.014). Morbidity mainly included bladder irregularities (6%), thickening (29%), and kidney dilation (6%). The G mutant allele on the rs3093660 marker was significantly associated with morbidity (χ^2^ = 4.47; *p* = 0.034; OR = 5.09 [95% CI: 1.04–24.9]). Our results suggest, for the first time, that carriers of the G mutant allele at rs3093660 marker have a five-fold increased risk of developing severe urogenital schistosomiasis.

## Abstract (fr)

Les facteurs génétiques de l’hôte influencent la gravité des maladies infectieuses, notamment la schistosomiase, qui représentent un problème majeur de santé publique en Afrique. Si le rôle du patrimoine génétique de l’hôte dans l’infection à *Schistosoma mansoni* est bien établi, ce lien reste peu exploré pour les infections à *S. haematobium* (Sh). Cette étude vise donc à examiner la relation entre le patrimoine génétique et la morbidité associée à la schistosomiase urogénitale par une approche de gènes candidats. Nous avons analysé des échantillons d’urine de 334 hommes béninois, en mesurant la protéine cationique éosinophile urinaire (ECP) par ELISA comme marqueur de l’inflammation vésicale. Une échographie abdominopelvienne a été réalisée chez un sous-groupe de 146 participants (69 Sh-positifs et 77 Sh-négatifs) afin d’évaluer les comorbidités associées à l’infection à *Schistosoma*. Des échantillons sanguins ont été analysés pour déterminer les taux de TNF-α par ELISA et les polymorphismes du promoteur du TNF-α par séquençage afin d’évaluer les associations entre la variation génétique et la morbidité. Les résultats ont montré que 25,4% des sujets *Sh*+ présentaient des taux moyens de TNF-α (U = 5888; *p* = 0,0098) et d’ECP (U = 912,5; *p* < 0,0001) significativement plus élevés que les sujets Sh−. Des corrélations positives ont été observées entre le nombre d’œufs et les taux d’ECP (Tau = 0,4016; *p* < 0,0001) ainsi que de TNF-α (Tau = 0,2238; *p* = 0,014). La morbidité comprenait principalement des irrégularités vésicales (6 %), un épaississement de la paroi vésicale (29 %) et une dilatation rénale (6 %). L’allèle mutant G du marqueur rs3093660 était significativement associé à la morbidité (χ^2^ = 4,47; *p* = 0,034; OR = 5,09 [IC à 95 % : 1,04–24,9]). Nos résultats suggèrent pour la première fois que les porteurs de l’allèle mutant G du marqueur rs3093660 présentent un risque cinq fois plus élevé de développer une schistosomiase urogénitale sévère.

## Introduction

Schistosomiasis is a major parasitic disease that mainly affects developing countries. In 2020, the World Health Organization (WHO) estimated that about 236 million people were at risk of this infection, with 90% in Africa [98]. In 2021, more than 151 million people were affected by schistosomiasis, with around 12,858 deaths reported worldwide [88]. Classified as a neglected tropical disease, schistosomiasis is the second most prevalent parasitic disease after malaria [19]. *Schistosoma haematobium* (*Sh*) and *Schistosoma mansoni* (*Sm*), responsible for the urogenital and mesenteric forms of schistosomiasis, respectively, cause the greatest burdens in Africa [34]. Since 2003, schistosomiasis control has focused on mass drug administration programs using praziquantel treatment for children aged 5 to 14 [95]. This treatment reduces prevalence by 91% and intensity of infection by 93% [95]. Despite this apparent effectiveness, schistosomiasis persists as a major health concern in several sub-Saharan African countries [11, 14, 57, 69]. The difficulty in controlling this parasitosis is partly due to the complexity of the parasite’s life-cycle which includes an initial environmental phase, as the disease is contracted through contact with fresh water contaminated by parasite larvae (cercariae) released by a snail vector [81]; and also a second phase that occurs in humans, where susceptibility to infection is influenced by environmental factors (such as density of infected snails and water temperature), socio-behavioral factors (such as frequency and modes of exposure to contaminated water, age, sex, and occupation), as well as factors specific to the human host, including immune response and genetic predispositions that affect resistance or susceptibility to severe forms of the disease [6, 20, 59, 90].

The influence of age, sex, exposure level, and immunity has been extensively studied in *Sm* infections. Studies have shown considerable differences in schistosome infection levels between individuals, even when infected in the same environment and exposed to similar frequencies [20, 32]. However, these factors account for only 20–25% of the variations between individuals [2, 65, 74]. Numerous studies have demonstrated that human genetic background significantly influences susceptibility to infection and the resulting morbidity. Mathematical models indicate that parasite distribution is highly aggregated, with approximately 80% of eggs released into the environment originating from only 20% of infected patients [102]. Genetic epidemiology studies have shown that 23% to 31% of the risk for *Sm* infection is due to human genetic factors [21]. The chromosomal region 5q31–q33, which includes several immune genes (*IL-4*, *IL-5*, *IL-9*, and *IL-13*), has repeatedly been associated with the intensity of infection, particularly with the number of eggs excreted in stools [21, 51, 64]. Other studies on the association between human host genetics and immune responses to helminths have demonstrated a strong correlation between single nucleotide polymorphisms (SNPs) in certain genes (*IFN-γ*, *IL-4, IL-10*…*etc.*) and immune responses to these parasites [29]. Thus, the analysis of single-nucleotide polymorphisms of immune genes appears to be a relevant approach to better understand the interactions between human host genetics and the severity of schistosomiasis. It has recently been reported that 32 candidate SNPs identified in 10 genes could help predict an individual’s susceptibility to developing severe mesenteric schistosomiasis with 63% sensitivity and 90% specificity [59]. Most of these genetic variants are located in genes involved in immunoregulatory functions, such as the *TNF-α* gene (encoding tumor necrosis factor-alpha, *TNF-α*) and the *RNASE3* gene (encoding eosinophil cationic protein, ECP). *TNF-α* is a potent pro-inflammatory cytokine that together with interferon-γ promotes granuloma formation, causing immunological pathologies in schistosomiasis [14]. *TNF-α* overexpression and functional variants located in the *TNF-α* promoter are implicated in numerous diseases and clinical parameters. For example, the *TNF-α* polymorphism rs1800629 has been associated with the severity of periportal fibrosis in *Sm* infected individuals in Brazil [89]. An association between infection intensity and polymorphism at the rs2073342 marker has been observed with respect to ECP [25]. Moreover, ECP also showed significant cytotoxic activity against *Sm* larvae, with differences in cytotoxicity depending on genetic variants [55, 56, 91].

In schistosome infection, pathology arises primarily from host immune responses directed against eggs trapped in tissues [14]. In morbidity studies of schistosomiasis [8, 58, 59], two major phenotypes are evaluated: (i) the intensity of infection, measured by the number of eggs excreted and/or by the detection of circulating antigens (cathodic circulating antigen in urine and anodic circulating antigen in plasma), and (ii) disease manifestations, including urinary tract lesions (kidney and ureter dilatation) for *Sh*, and liver lesions (periportal fibrosis, portal hypertension) for *Sm* [30, 57, 70]. These disease manifestations are detected by ultrasound examinations [79, 80].

Human genetics is known to influence *Sm* infections, but this is less well studied in *Sh* infections despite their significant medical implications. Importantly, *Sh* is the only schistosome species classified as carcinogenic, due to its established association with bladder cancer [13, 35]. In addition, chronic infection frequently results in egg deposition in the genital organs (bladder, cervix) [44, 75], seminal vessels, and prostate [45], which can lead to sterility in adulthood. Furthermore, *S. haematobium* may contribute to HIV transmission by increasing viral load in semen and inducing hemorrhagic lesions in the cervical mucosa of infected women [60]. In this context, the present study aimed to explore the impact of human genetic background on morbidity associated with bladder and kidney diseases induced by *Sh* infection. The study focused on immune genes in young men from rural areas in Benin with high schistosomiasis endemicity, specifically the urogenital form. Understanding the influence of genetic factors in the susceptibility and severity of this infection could pave the way for new prevention, treatment, and vaccination strategies [43]. We examined the correlation between the infection levels, the morbidity-related disease manifestations assessed, and the single-nucleotide polymorphisms (SNPs) in the *TNF-α* and ECP candidate genes. Additionally, studies have shown the roles that these SNPs play in the severity of periportal fibrosis [89] and the intensity of infection [25] in *Sm* infection. In line with the new WHO roadmap for the elimination of schistosomiasis as a public health problem by 2030 [97], this study provides essential data on the interactions between the genetic diversity of the human host and the morbidity associated with *Schistosoma* spp*.* infections.

## Materials and methods

### Ethics approval

The study was approved by the Comité National d’Éthique pour la Recherche en Santé (CNERS) of the Benin Ministry of Health under authorization reference 063/MS/DC/SGM/CNERS/SA of March 30, 2022. Written consent was obtained from village chiefs and all participants. The parents of patients under 18 years of age were informed and we obtained their oral consent. In cases where those who had to give permission could not read or write, a detailed verbal explanation of the form was given in order to obtain informed consent. Two copies of the written consent form were signed and dated. One copy was retained by each patient, and the second copy was archived in our database. Parasitological and pathological status was communicated to all participants found to have schistosomal infections or other diseases, such as prostatic hypertrophy. Those infected with schistosomiasis were treated with praziquantel (40 mg/kg body weight), in accordance with WHO recommendations [94]. Patients with infections other than schistosomiasis were referred to health centers for more appropriate treatment.

### Study sites

**Figure 1:**
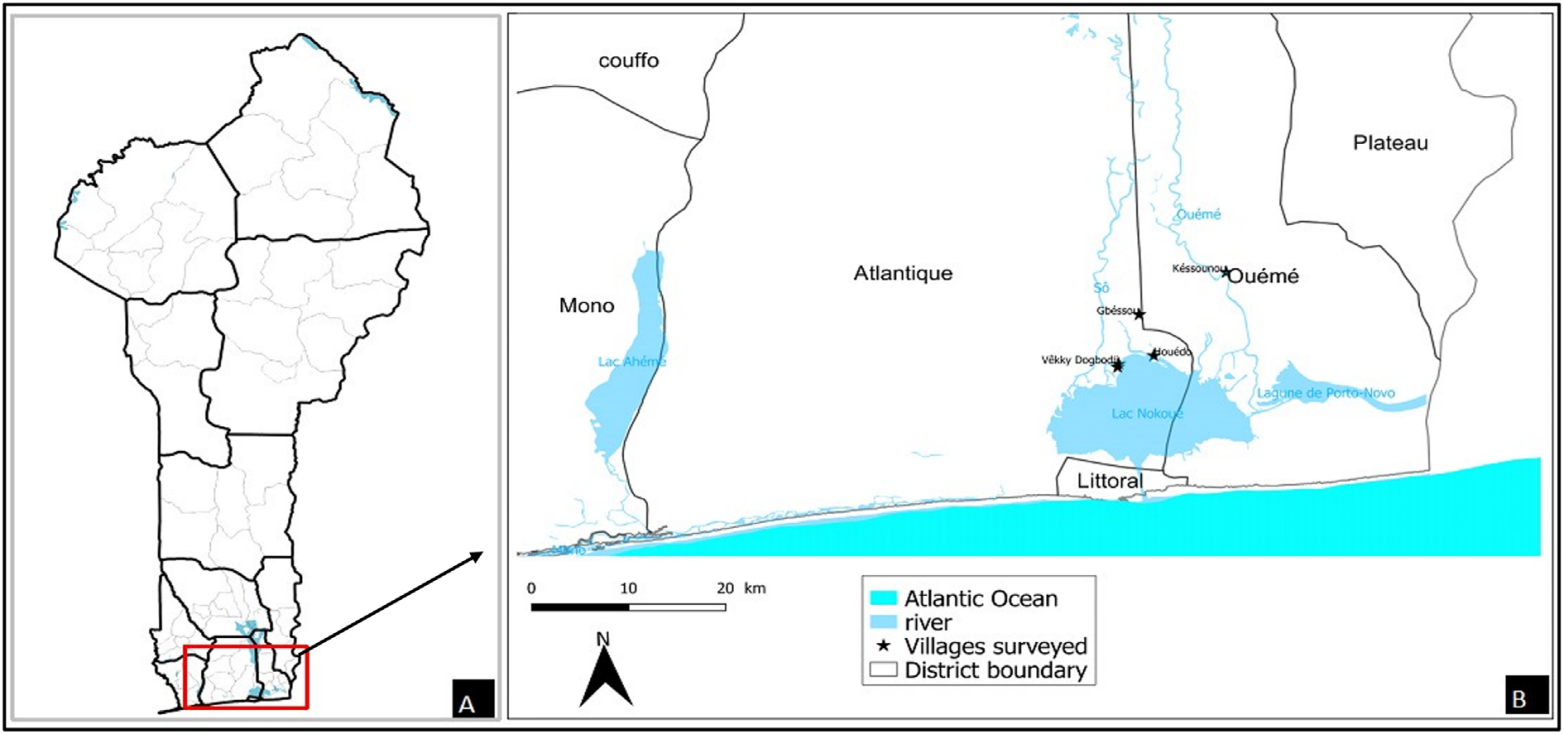
Map of the study area showing villages sampled during the survey. A) Geographical situation of Benin. B) Localization of main villages surveyed.

This study was carried out in two communes (Sô-Ava and Dangbo) in southern Benin (Fig. 1), where urogenital schistosomiasis transmission is active [85, 86]. Cross-sectional surveys were conducted between March and May 2022 among young male volunteers aged 14 and older in the lakeside villages of Gbéssou, Vêkky, and Houédo (Sô-Ava district) and Késsounou (Dangbo district). These villages are characterized by a sub-equatorial climate with four distinct seasons: (i) a long rainy season from March to June, (ii) a short dry season from July to August, (iii) a short rainy season from September to October, and (iv) a long dry season from November to February.

### Sample collection

**Figure 2:**
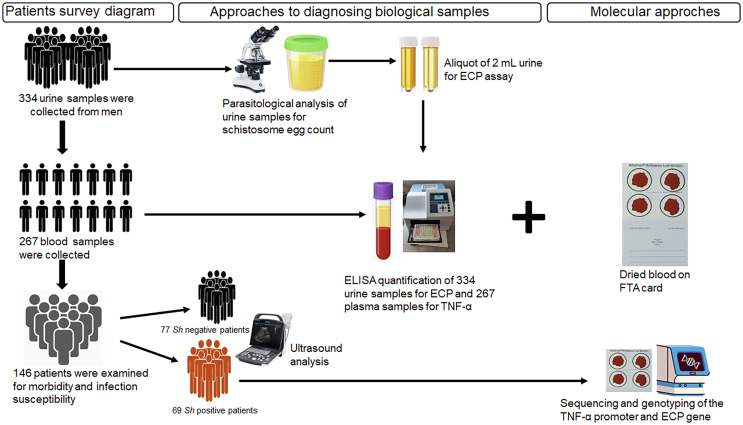
Summary of study design.

The sample collection protocol is described in Figure 2. Urine samples were collected from 334 participants in 250 mL containers, and 267 of them also provided approximately 2 mL of venous blood in EDTA tubes. The samples were collected between 10:00 am and 14:00 pm and transported to the laboratory in a refrigerated cooler. For each participant, about 5 mL of homogenized urine was aliquoted and stored at –80 °C within four hours of collection for subsequent measurement of eosinophil cationic protein (ECP). The remaining urine was processed using the filtration method to assess urogenital schistosomiasis infection [73].

Blood plasma was extracted from each sample after centrifugation and was stored at −80 °C to quantify TNF-α levels. Fifty microliters of the blood pellet were then dried using FTA cards for *TNF-α* and *ECP* genotyping. Among the participants examined for *Schistosoma* infection, 146 (including all *Sh* positive patients (*n* = 69)) invited participants underwent abdominal-pelvic ultrasound examination.

### Urogenital schistosomiasis diagnostic

The urine filtration technique was employed using a 10 mL syringe and a filtration device containing a 13 mm diameter polycarbonate membrane with 12 μm pore size (Polycarbonate (PC) hydrophilic, it4ip, Louvain-la-Neuve, Belgium). For each sample, 10 mL of homogenized urine was drawn with a sterile syringe and passed through the membrane. The membrane was then carefully removed with forceps, placed on a clean glass slide, and stained with a drop of Lugol’s iodine solution. *Schistosoma* eggs were subsequently counted by microscopic examination.

### ECP quantification

Urinary eosinophil cationic protein (ECP) levels were measured using a standard enzyme-linked immunosorbent assay with the MESACUP ECP TEST kit (MBL International, Woburn, MA, USA) [5]. One hundred microliters of prepared urine samples (50 μL of urine sample to 200 μL of assay diluent) were transferred to a 96-well microplate pre-coated with anti-human ECP antibody. After incubation and washing, 100 μL of horseradish peroxidase conjugated anti-human ECP polyclonal antibody was added, followed by tetramethylbenzidine/H_2_O_2_ substrate. The absorbance was read at 450 nm using an infinite 200Pro enzyme-linked immunosorbent assay (ELISA) microplate reader (Tecan, Männedorf, Switzerland) and results were interpreted according to the protocol provided by the manufacturer. We also ran the positive and negative controls supplied in the kit in triplicate. Additionally, the results for each plate were validated in accordance with the kit manufacturer’s specifications: a 450 nm absorbance reading of ≤ 0.2 for standard 1 and ≥ 1.2 for standard 6.

### TNF-α quantification

Plasma TNF-α levels were measured using a standard ELISA with Invitrogen’s Human TNF-α uncoated kit, following the manufacturer’s protocol. Microtiter plates were coated with 100 μL of anti-human TNF-α antibody, previously diluted according to the manufacturer’s instructions, then incubated overnight at 4 °C. After washing, wells were blocked with the kit’s blocking buffer for 1 hour at room temperature. Serial dilutions of standard TNF-α (recombinant human TNF-α) and samples (patient plasma) were added (100 μL/well), followed by incubation at room temperature for two hours. After a further wash, biotin-conjugated anti-human TNF-α detection antibody and streptavidin-HRP were added. The yellow color obtained after stopping the reaction with tetramethylbenzidine substrate was read at 450 mm in an infinite 200Pro ELISA microplate reader (Tecan).

### Assessment of morbidity by ultrasound

A subgroup of patients, both infected (*Sh*+) and uninfected (*Sh−*), who provided urine and blood samples were examined by ultrasound. This was done to assess lesions caused by the infection in the urinary tract and at the hepatosplenic level, in order to correlate infection levels with ureteral, renal, and intestinal disorders. The presence of urinary tract and intestinal disorders was determined using a portable ultrasound machine (DP-30, Mindray, Shenzhen, China) equipped with 3.5 MHz convex transducers. The ultrasonographer was unaware of the status of the tested subjects. Half an hour before the ultrasound examination, each patient was given approximately 500 mL of water. The examination was performed only when the bladder was completely full. Subjects were examined in the supine position with the transducer positioned to obtain the best image. Images were recorded and scored for renal size, hydronephrosis, hydroureter, bladder thickening, and bladder irregularities to assess urinary tract disease, while liver, spleen, and abdominal vessel size were scored for bowel disease. All ultrasound image analyses were performed according to the standardized Niamey protocol [79, 80]. The ultrasound data allowed us to determine whether a participant had urinary tract disease by summing the scores as recommended by the WHO according to degree [79, 80]. Participants with a total score of zero (score = 0) and those with a score of at least 1 (score ≥ 1) were classified as having urogenital schistosomiasis with morbidity undetectable by ultrasound (IMU) and with morbidity detectable by ultrasound (IMD), respectively. Other indicators such as the liver image compared with liver patterns A, B, C, D, E, F, X, Y, and U showing the extent of fibrosis or other parenchymal pattern [80], measurements of left and right hepatic lobe size and portal vein internal diameter were adjusted to the patient’s height to assess intestinal lesions. Following WHO guidelines, periportal wall measurements were obtained by assessing the external and luminal diameters of the widest segmental portal branch, measured as near as possible to the branch point [80]. The internal diameter of the portal vein near its entrance into the liver was also measured, and the average of the two measurements was calculated to identify any hepatic periportal thickening. The evaluation of fluid accumulation in the abdomen, known as ascites, as well as the measurements of the sizes of both liver lobes and the portal vein diameter, were used to assess portal hypertension in the patients. Additionally, the height of each patient was measured precisely to the nearest centimeter using a fixed tape measure. Based on the obtained measurements, an index was calculated by dividing the mean participant height by the diameter of the periportal wall, the portal vein, or the size of both liver lobes, in order to correct for patient size differences before assigning scores, as recommended by the WHO [80]. The scores obtained for the portal vein and liver lobes were combined with a score for the presence or absence of ascites to yield a final score for assessing the presence of portal hypertension in the participants. A final score of zero indicates the absence of hepatic portal thickening or portal hypertension.

### *ECP* and *TNF-α* polymorphism

#### DNA extraction

We used FTA-card-dried blood samples from patients found to be infected with urogenital schistosomiasis. Genomic DNA was extracted using a QIAamp DNA mini kit (QIAGEN, Hilden, Germany), in accordance with the manufacturer’s instructions.

#### ECP genotyping

The rs2073342 ECP polymorphism in the ECP gene, located on chromosome 14, was targeted by the restriction fragment length polymorphism (RFLP-PCR) method as previously described [25, 38, 71]. Primers 5′–GTGTGTCATAACCGAGACCGGATAG–3′ and 5′–GGACAGTTGCTGATACCCAGAGTAC–3′ [38] were used for the amplification reaction. Amplification was carried out in a final volume of 25 μL, containing 2 μL of DNA, 1.5 μL of 25 mM MgCl2, 5 μL of 5× buffer (Promega, Madison, WI, USA), 1 μL of each of the 10 μM primers, 0.5 μL of 10 mM dNTP, 0.2 μL of Go Taq polymerase (Promega) and 13.8 μL of milli-Q water. Each cycle included a DNA denaturation step at 95 °C for 40 s, followed by a primer hybridization step at 51 °C for 40 s and an elongation step at 74 °C for 1 min 10 s. The program continued and ended with a final extension step at 74 °C for 5 min. In the next step, 17 μL of PCR products were digested at 37 °C for 15 min using 0.1 μL 10 U PStI restriction enzyme (New England Biolabs, Ipswich, MA, USA), 5 μL of CutSmart buffer, and 27.9 μL of water, in a total reaction volume of 50 μL. The reaction was then inactivated at 80 °C for 20 min. The PStI enzyme cut sites for the ECP-specific SNP rs2073342 were based on the sequence positions 5′–CTGCA↓G–3′ and 3′–G↑ACGTC–5′. This enzymatic digestion cuts the 644 bp ECP fragment into three bands for the heterozygous ECP +434 GC genotype (644 bp, 430 bp, and 214 bp); two bands for the homozygous ECP +434 GG genotype (430 bp and 214 bp), and a single band for the homozygous ECP +434 CC genotype (214 bp) [71]. Digestion of PCR products from the 76 samples that successfully amplified the ECP gene was visualized using 2% agarose electrophoresis gels stained with GelRed^TM^ (Biotium Inc. Darmstadt, Germany). Sequencing confirmed 30 profiles to validate the band profiles obtained from the gels.

### TNF-α genotyping

#### Primer design

To analyze SNPs in the promoter region of the TNF-α gene, two primer pairs were designed using Geneious software, and based on DNA sequences (accession No. NG_007462.1). The primer sequences were as follows: (i) BS_TNF1F: 5′–ATCTGCACCCTCGATGAAGCC–3′ and BS_TNF1R: 5′–ATCTGCACCCTCGATGAAGCC–3′ (amplicon size: 1115 bp); (ii) BS_TNF2F: 5′–ATCAGTCAGTGGCCCAGAAGACC–3′ and BS_TNF2R: 5′–CACCTTCCAGGCATTCAACAGC–3′ (amplicon size: 1,116 bp). Primer specificity was validated using NCBI’s Primer-BLAST, ensuring alignment with the target gene. Furthermore, Sigma Oligoanalyser (https://www.oligoevaluator.com) was used to evaluate potential hairpins, self-dimers, and heterodimers. These two primer pairs collectively covered a 2,122 bp region of the *TNF-α* gene promoter, allowing comprehensive genotyping of SNPs within the promoter.

#### DNA amplification and sequencing

For the BS_TNF1F/BS_TNF1R primer pair, the amplification conditions included the activation phase at 95 °C for 5 min, followed by 45 amplification cycles. Each cycle involved a DNA denaturation step at 95 °C for 45 s, followed by a primer hybridization step at 51 °C for 45 s, and an elongation step at 72 °C for 1 min 10 s. The program continued and ended with a final extension phase at 72 °C for 7 min. Amplification conditions for the BS_TNF2F/BS_TNF2R pair consisted of an activation phase at 96 °C for 5 min, followed by 45 amplification cycles. Each cycle included a DNA denaturation step at 96 °C for 45 s, followed by a primer hybridization step at 58 °C for 45 s, and an elongation step at 74 °C for 1 min 10 s. The program continued and ended with a final extension step at 74 °C for 7 min. All PCR products successfully amplified for both primer pairs were purified and sequenced with primers BS_TNF1R: 5′–ATCTGCACCCTCGATGAAGCC–3′ and BS_TNF2R: 5′–CACCTTCCAGGCATTCAACAGC–3′, respectively on an Applied Biosystems genetic analyzer at Genoscreen (Lille, France).

#### Sequence analysis

Successfully sequenced DNA sequences were assembled and manually edited using Sequencer, version 4.5 (Gene Codes Corporation; http://genecodes.com) to remove ambiguities and sequencing errors. The cleaned sequences were aligned using MUSCLE implemented in MEGA, version 7.0.26 [22, 42]. The two partial *TNF-α* sequences (TNF1 and TNF2) obtained from the same individual were concatenated using Geneious version 4.8.5 (www.geneious.com) to generate a single consensus sequence. The *TNF-α* gene is a bi-allelic autosomal gene located on chromosome 6. We used the PHASE module of the DnaSP 6.12.03 program [83] to generate the two allelic sequences for each patient. The program generated two sequences for each of the input sequences, giving a total of 156 sequences for the 78 *Sh*+. The Haploview program [7] was used to assess genetic linkage between pairs of markers associated with observed morbidity through linkage disequilibrium (LD) analysis. A block matrix was generated based on the degree of linkage using the *R*^2^ value (expressed as a percentage), with each number indicating the level of association between marker pairs. To determine whether the distribution of alleles for each SNP conformed to the Hardy-Weinberg equilibrium expectations, and to perform a case-control association analysis (IMD vs IMU), a chi-squared contingency table was constructed and the corresponding values were calculated.

### Statistical analysis

A Shapiro test was used to assess the normality of ECP, TNF-α, and *Sh* infection intensity data. ECP and TNF-α showed a positively skewed distribution. To normalize the distribution and reduce the impact of extreme values, a log10 transformation was applied before statistical analysis. A Kruskal–Wallis test, followed by *post-hoc* pairwise comparisons using Dunn’s test with Bonferroni adjustment, was also used to assess whether disease status was associated with differences in parasite intensity (egg count), ECP, and plasma TNF-α concentrations.

Differences in immune protein levels (ECP and TNF-α) between patient groups (infected and uninfected) and between marker genotypes were compared using the a Wilcoxon–Mann–Whitney and Kruskal–Wallis tests. Kendall’s correlation was used to determine the association between the mean *Schistosoma* egg count for *Sh* and the level of immune proteins (ECP and *TNF-α*). Chi-square tests with Monte Carlo approximation were used to analyze the distribution of genotypes of markers with three variants between the IMD and IMU groups. Of note, the data were structured in the form of a 2 × 3 contingency table, where the rows represent the groups (IMD and IMU) and the columns the different genotypes. Considering the presence of small numbers for some genotypes, we applied a Monte Carlo approximation with 10,000 simulations in order to obtain a more robust estimate of the *p*-value. The analysis was performed using the *chisq.test*() function in R software, with the *simulate.p.value* option activated to compensate for the small numbers and ensure the validity of the independence test.

A generalized linear model (GLM) was used to perform ANOVA (analysis of variance) to test the effect of genetic variants on the *S. haematobium* egg count/intensity, and pairwise comparison groups were evaluated using Tukey’s test. The association of alleles with morbidity was also measured using the Chi-square test. Univariate statistical analyses were performed using BiostaTGV (https://biostatgv.sentiweb.fr) and R software. The test was considered significant when the *p*-value was less than 0.05.

## Results

### Prevalence of *S. haematobium* and egg count

Among the participants, 25.4% (85/334) had *Sh* eggs in their urine. The mean *Schistosoma* egg count ± standard error of *Sh* eggs per 10 mL of urine was 16.74 ± 3.27 with the minimum and maximum counts of 1 and 193 eggs/10 mL, respectively.

#### ECP and TNF-α concentrations in urine and blood

**Figure 3:**
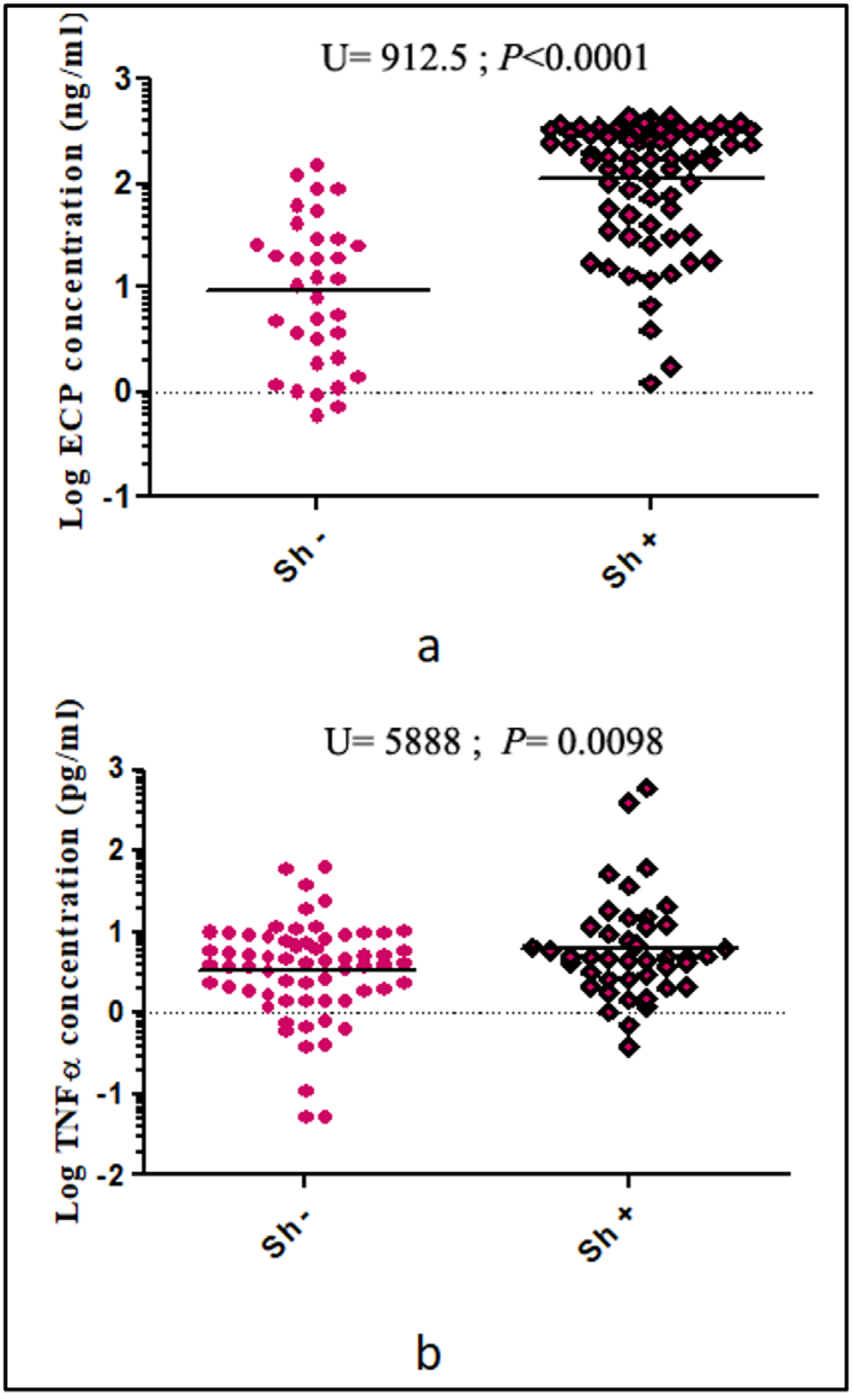
Levels of ECP (a) and TNF-α (b) in urine and blood of patients with (*Sh*+) or without (*Sh*−) *S. haematobium* infection. Horizontal bars indicate the geometric means for each group. Log transformations have been used due to the inherent differences in the data for each biomarker (ECP and TNF-α).

Mean urinary ECP levels were significantly higher in *Sh*+ than in *Sh*− (183.4 ± 16.7 ng/mL *vs.* 3.9 ± 1.3 ng/mL, U = 912.5; *p* < 0.0001). Similar observations were recorded for mean plasma levels of TNF-α (17.6 ± 9.1 pg/mL *vs.* 2.5 ± 0.6 pg/mL, U = 5888; *p* = 0.0098) (Fig. 3a and 3b). Furthermore, there was a significant Kendall correlation between the number of eggs present in urine and ECP levels (Tau = 0.40; *p* < 0.0001) and TNF-α (Tau = 0.22; *p* = 0.014) observed in urine and blood, respectively.

#### Urinary tract disorders detected on ultrasound

**Figure 4:**
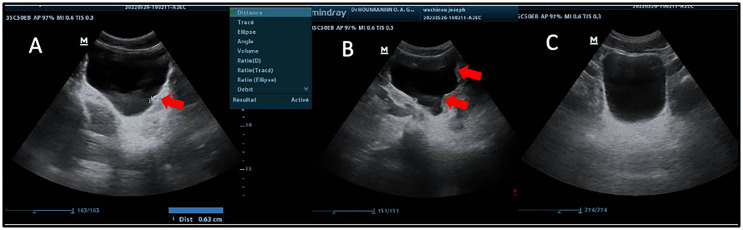
Ultrasound images showing some lesions observed in the bladder of patients with (*Sh*+) or without (*Sh*−) *S. haematobium* infection. A: Bladder of irregular shape and thickened wall (red arrow); B: Bladder of irregular shape with pseudopolyps (red arrow); C: Normal bladder; M: Male patient.

Among the 146 patients examined by ultrasound, 69 (47%) were *Sh*+ and 77 (53%) were *Sh*−. The prevalence of urinary tract disorders associated with *Sh*+ was significantly higher (Fisher Test; *p <* 0.0001) (36.23%) than in *Sh*− (7.80%) (Table 1). Our results show that disorders of the bladder (lower urinary tract) were much more frequent than those of the upper urinary tract. Regarding lower urinary tract disorders, bladder thickening was the most frequent (Fig. 4), which accounted for 21 patients (14.38%), followed by bladder irregularities (focal or multifocal) in 8 patients (5.48%). Severe bladder disorders such as masses and polyps were rare, with only one case observed. The prevalence of bladder wall irregularity appeared to be the same in both groups, despite the observed bladder wall thickening, which was significantly more prevalent in *Sh*+ cases than in *Sh*− (Fisher Test; *p <* 0.0001); the prevalence of irregularity appeared to be the same in both groups. As for upper tract disorders, they were observed in the *Sh*+ group only (Table 1). Dilatation of the kidneys and ureters was observed in four (2.74%) and three (2.05%) patients respectively.

**Table 1:** Ultrasound lesion findings in patients with (*Sh+*) and without (*Sh−*) *S. haematobium* infection. The measurements in mm are the size of bladder wall thickening.

Observed morbidity	Examined (*n* = 146)	*Sh+* (*n* = 69)	*Sh−* (*n* = 77)	
Disease detected on ultrasound	21.23% (31)	36.23% (25)	7.80% (06)	*p <* 0.0001
Bladder disease				
Abnormal shape	4.79% (07)	2.90% (02)	6.49% (05)	NS
Irregular shape	5.48% (08)	5.80% (04)	5.19% (04)	NS
Thickening	14.38% (21)	28.99% (20)	1.30% (01)	*p <* 0.0001
Weights	0.68% (01)	0% (00)	1.30% (01)	NA
Pseudopolyp	0.68% (01)	1.45% (01)	0% (00)	NA
Upper urinary tract disease				
Hydroureter	2.05% (03)	4.35% (03)	0% (00)	NS
Hydronephrosis	2.74% (04)	5.80% (04)	0% (00)	NS
Assessment of bladder wall thickening				
Normal wall (< 5 mm = Grade 0)	84.93% (124)	71.01% (49)	97.40% (75)	*p <* 0.0001
Mild thickening (5–7 mm = Grade 1)	7.53% (11)	14.49% (10)	1.30% (01)	*p =* 0.003
Moderate thickening (8–9 mm = Grade 2)	6.16% (9)	13.04% (9)	00% (00)	*p =* 0.0009
Severe thickening (≥10 mm = Grade 3)	1.37% (2)	1.45% (1)	1.30% (01)	NS

NS = not significant; *n* = sample number; NA = not applicable.

The overall severity index scores recorded from the ultrasound ranged from 0 to 20, which enabled the classification of observed conditions into five categories: no disease (score 0), mild (score 1–2), moderate (score 3–4), severe (score 5–6), and very severe (score ≥ 7) (Table 2). Analysis of the morbidity scores observed from ultrasound showed that the severity index was higher and increased (score 0 to score 20) in the *Sh*+ group compared to the *Sh*− group (Table 2). The higher frequency of zero morbidity scores as observed in *Sh*− group revealed the absence of urinary disease.

**Table 2:** Overview of the distribution of morbidity scores measured by ultrasound in patients with (*Sh*+) and without (*Sh*−) *S. haematobium* infection.

	Urinary tract score	Total	Score 0	*p*	Score 1 to Score 2	*p*	Score 3 to Score 4	*p*	Score 5 to Score 6	*p*	Score ≥ 7	*p*
Patients examined by ultrasound	Number of *Sh*−	77	71	<0.0001	04	0.0008	01	0.005	01	NS	00	0.04
	Number of *Sh*+	69	41		17		06		01		04	
Ultrasound morbidity in *Sh*+ patients	Number of IMU	41	41		00		00		00		00	
	Number of IMD	28	00		17		06		01		04	
	Disease level		No disease (Normal)		Mild urinary disease		Moderate urinary disease		Severe urinary disease		very severe urinary disease	

NS = not significant; *p* = Fisher test *p*-value; *Sh*+: patients infected with *Sh; Sh*−: patients uninfected with *Sh*; IMU: infected with morbidity undetected on ultrasound; IMD: infected with morbidity detected on ultrasound.

Concerning lower urinary tract disorders, the observed lesions included abnormal bladder shape, wall irregularities, bladder thickening, weights, and pseudopolyps (Table 1). The first three were more frequent than the last two, with bladder thickening significantly more prevalent in the *Sh*+ group than in the *Sh*− group (Fisher test; *p* < 0.0001). Bladder thickening was classified into four grades, *i.e.*, from 0 to 3 (See Table 1). Most patients in the *Sh*− group had a normal bladder without thickening (Fisher test; *p* < 0.0001), except for two cases with mild and severe thickening, respectively. Mild (Fisher test; *p* = 0.003) and moderate (Fisher test; *p* = 0.0009) bladder thickening were significantly more frequent in the *Sh*+ group. Upper urinary tract disorders, including ureteral and renal dilation were only observed in the *Sh*+ group.

#### Hepatic disorders detected on ultrasound

Hepatic fibrosis was observed in only one patient with image template D in reference to the Niamey protocol [80]. All other liver images were classified as normal (image template A). No hepatic periportal thickening or portal hypertension were detected in either groups examined (*Sh*+ and *Sh*−).

### Relationship between urinary tract morbidity and levels of ECP, TNF-α, and parasite intensity

**Figure 5:**
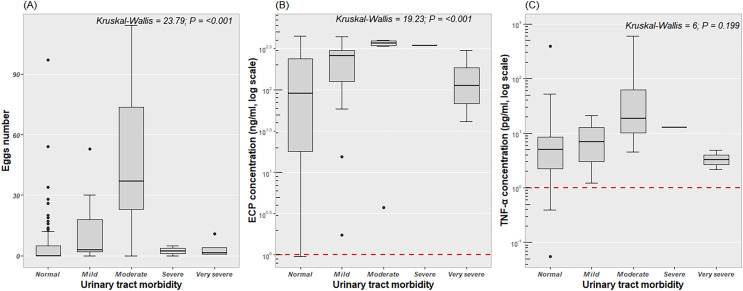
Relationship between severity of disease (morbidity), ECP levels (A), TNF-α levels (B), and parasite intensity (C) in patients infected with *S*. *haematobium*. Score 0 = No disease (normal); Score 1–Score 2 = Mild urinary disease; Score 3–Score 4 = Moderate urinary disease; Score 5–Score 6 = Severe urinary disease; Score ≥ 7 = very severe urinary disease.

The Kruskal–Wallis test revealed significant differences across disease groups in egg counts (KW = 23.80; *p* < 0.001) and ECP levels (KW = 19.23; *p* < 0.001) but not in TNF-α concentrations (KW = 6; *p* = 0.2) (Figs. 5A to 5C). *Post-hoc* Dunn’s tests also showed that both the mild and moderate groups had significantly higher egg counts and ECP levels compared to other group (*p* < 0.05). Urinary ECP levels and the number of eggs counted increased with mild and moderate morbidity, but decreased when morbidity was classified as normal, severe, and very severe.

IMD patients (218.27 ± 30.08 ng/mL) had a significantly higher ECP level (*W* = 1,711; *p* < 0.0001) than IMU patients (145.83 ± 22.65 ng/mL). Although an elevated mean *TNF-α* level could be observed in IMD patients (28.69 ± 22.51 pg/mL) compared to IMU patients (14.96 ± 10.49 pg/mL), we were unable to statistically prove this difference (*W* = 51; *p* = 0.2763).

### ECP and *TNF-α* gene polymorphisms and their association with the disorder

**Figure 6:**
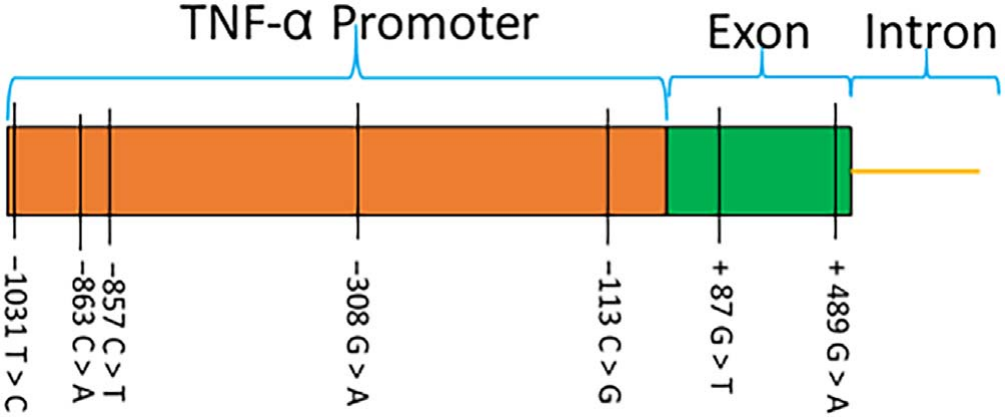
Schematic representation of the 2,122 bp promoter and fragment of the sequenced *TNF-α* gene, showing the location of the various SNPs.

The genotype frequencies of each marker present on the *ECP* and *TNF-α* genes are presented in Table 3. The rs2073342 GC and rs2073342 CC genotypes of the ECP gene were the most common among patients. None of the rs2073342 marker genotypes showed significant differences between the IMD and IMU groups (*χ*^2^ = 0.98; *p =* 0.71) (Table 3). For the *TNF-α* gene, sequencing of the promoter and a gene fragment showed the presence of (i) five point mutations upstream of the transcription start site, and (ii) two mutations in the first exon (Fig. 6). All seven mutations have previously been reported in other studies, and all have dbSNP identification numbers (−1031 T > C (rs1799964), −863C > A (rs1800630), −857C > T (rs1799724), −308 G > A (rs1800629), −113 C > T/G (rs3093660), +87 G > T (rs2228088), and +489 G > A (rs1800610)). Comparison of the genotype distribution of markers on the *TNF-α* gene showed no strong evidence of a significant difference between the IMD and UMD groups (Table 3).

**Table 3:** *ECP* and *TNF-α* SNP distribution in patients infected with *S. haematobium* according to the morbidity level revealed by ultrasound (IMD or IMU).

Genes	*ECP*			*TNF-α*																			
Name SNP	+434 G > C			−1031 T > C			−863 C > A			−857 C > T			−308 G > A			−113 C > G		+87 G > T			+489 G > A		
(dbSNP ID number)	(rs2073342)			(rs1799964)			(rs1800630)			(rs1799724)			(rs1800629)			(rs3093660)		(rs2228088)			(rs1800610)		
Genotypes	CC	GG	GC	CC	TT	TC	CC	AA	AC	CC	TT	TC	AA	GG	AG	CC	GG	GG	TT	GT	AA	GG	AG
All (%)	34.2	17.1	48.7	5.2	84.5	10.3	80.6	3.2	16.2	95.2	1.6	3.2	3.1	76.6	20.3	93.2	6.8	90.4	1.4	8.2	2.7	91.8	5.5
	*N* = 76			*N* = 58			*N* = 62			*N* = 62			*N* = 64			*N* = 73		*N* = 73			*N* = 73		
IMD (%)	34.6	23.1	42.3	5.9	76.5	17.6	70	0	30	100	0	0	4.6	63.6	31.8	84.6	15.4	84	00	16	00	96	4
	*N* = 26			*N* = 17			*N* = 20			*N* = 20			*N* = 22			*N* = 26		*N* = 25			*N* = 25		
IMU (%)	40.5	13.5	46	6.5	93.5	0	84.4	6.2	9.4	93.8	3.1	3.1	3.1	81.3	15.6	97.3	2.7	91.9	2.7	5.4	2.7	91.9	5.4
	*N* = 37			*N* = 31			*N* = 32			*N* = 32			*N* = 32			*N* = 37		*N* = 37			*N* = 37		
	χ^2^ = 0.98 ; *P* = 0.71			χ^2^ = 5.84 ; *p* = 0.07			χ^2^ = 4.60 ; *p* = 0.09			χ^2^ = 1.3 ; *p* = 1			χ^2^ = 2.16 ; *p* = 0.37			Fisher test; *p* = 0.30		*p* = 0.27; χ^2^ = 2.51			*p* = 1 ; χ^2^ = 0.76		

IMU = infected with morbidity undetected on ultrasound; IMD = infected with morbidity detected on ultrasound; *TNF-α*: tumor necrosis factor-alpha; ECP: eosinophil cationic protein, *N* = sequences number; *p* = statistical *p*-value; χ^2^ = Chi square.

Adjustment of the data using the linear regression model (GLM) showed that the genetic polymorphisms observed in the *ECP* and *TNF-α* genes had a significant effect on the number of eggs observed, except for the rs1800629 marker (Table 4). Tukey’s *post-hoc* test showed that patients with genotype rs2073342 GG for the ECP gene and rs1799964 CC; rs1800630 AA; rs1799724 TC; rs3093660 GG; rs2228088 GG for the *TNF-α* gene were associated with a significantly higher number of eggs in the urine compared to the other genotypes found on the same markers (Supplementary file 1). The level of the ECP immune protein was significantly higher in patients with the GG genotype than in those with the CC genotype for the rs3093660 marker (*W* = 75; *p =* 0.04) (Supplementary file 2). No significant differences were observed between genotypes for the other ECP markers. There were also no significant differences in plasma *TNF-α* levels between genotypes (Supplementary file 2).

**Table 4:** Summary of the generalized linear model output to determine the effect of *TNF-α* and *ECP* polymorphisms on eggs counts.

SNP name	dbSNP ID number	*N*	df	Deviance	Resid. Df	Resid. Dev	*F*	Pr(>F)
+434 G > C (*ECP*)	rs2073342	76	2	76.70	73	2112.80	38.35	< 0.0001
**−**1031 T > C	rs1799964	58	2	50.66	55	1783.20	25.33	< 0.0001
**−**863 C > A	rs1800630	62	2	105.21	59	1806.80	52.61	< 0.0001
**−**857 C > T	rs1799724	62	2	21.737	59	1890.3	10.869	< 0.0001
**−**308 G > A	rs1800629	64	2	5.44	61	1968.20	2.72	0.066
**−**113 C > G	rs3093660	73	1	321.29	71	1805.30	321.29	< 0.0001
+87 G > T	rs2228088	73	2	92.22	70	2034.40	46.11	< 0.0001
+489 G > A	rs1800610	73	2	13.328	70	2113.3	6.6641	0.0013

*N* = sample number; df = degrees of freedom; Resid. Df = residual degrees of freedom; Resid. Dev = residual deviance; F = F-statistic; Pr(>F) = *p*-value for the F-test.

**Figure 7:**
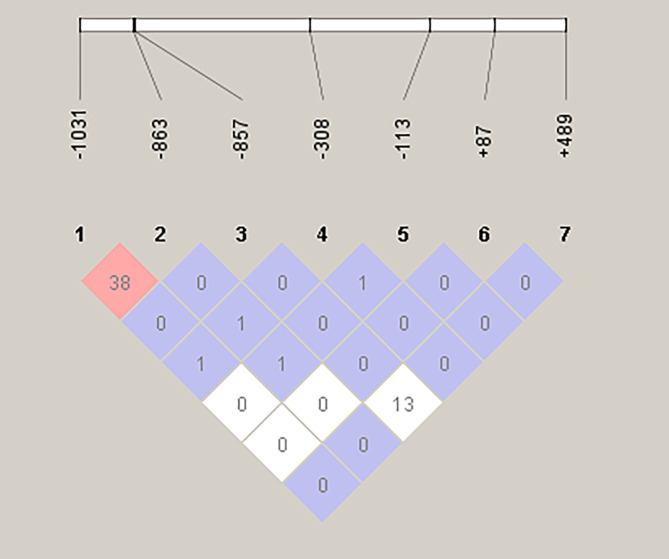
Tests for pair-wise linkage disequilibrium tests between SNPs in *Sh* infected groups in relation to morbidity. The numbers in the boxes are the *R*^2^ (%) values. The stronger the association between two SNPs, the higher the *R*^2^ value.

Linkage disequilibrium (LD) analysis between marker pairs located in the *TNF-α* gene revealed a weak correlation between rs1799724 and rs1800610 (*R*^2^ = 0.13), and a moderate correlation between rs1799964 and rs1800630 (*R*^2^ = 0.38). For all other marker pairs, no correlation (*R*^2^ = 0) or a negligible level of association (*R*^2^ = 0.01) was observed (Fig. 7). A statistically significant deviation from expectations under the Hardy-Weinberg equilibrium could be detected in *TNF-α* mutations at positions −1031, −113, and +489. Allelic case-control (IMD vs IMU) association analysis revealed that only the G mutant allele at position -133, located on marker rs3093660 within the *TNF-α* gene, was significantly associated with morbidity (Table 5). Our findings show that the distribution of TNF-α alleles differed significantly between IMD patients and IMU (*χ*^2^ = 4.47, *p* = 0.034). The G allele was more frequent among IMD patients (15.4%) than among IMU patients (3.4%), corresponding to an odds ratio of 5.09 [95% CI: 1.04–24.9]. The C allele on this marker (rs3093660) seemed not to be linked to the severity of infection.

**Table 5:** Test for association of *TNF-α* gene alleles with morbidity and Hardy–Weinberg equilibrium in patients infected with *S. haematobium*.

No.	SNP Name	dbSNP ID number	SNP positions on GRCh38	Allele associated	IMD Frequency	IMU Frequency	Chi square (χ^2^)	*p*-value χ^2^	HW *p*-value
1	**−**1031 T > C	rs1799964	31574531	C	0.147	0.065	1.761	0.1845	0.0173
2	**−**863 C > A	rs1800630	31574699	A	0.150	0.109	0.371	0.5422	0.3106
3	**−**857 C > T	rs1799724	31574705	C	1.000	0.953	1.931	0.1647	0.0972
4	**−**308 G > A	rs1800629	31575254	A	0.205	0.109	1.871	0.1713	0.579
5	**−**113 C > G	rs3093660	31575629	G	0.130	0.029	4.486	0.0342	4.5467E-8
6	+87 G > T	rs2228088	31575828	T	0.087	0.043	0.952	0.3292	0.2953
7	+489 G > A	rs1800610	31576050	G	0.978	0.971	0.051	0.8206	0.2141

NA = not available; GRCh38 = genome reference consortium human build 38; IMU = infected with morbidity undetected on ultrasound; IMD = infected with morbidity detected on ultrasound; SNP = single nucleotide polymorphism; HW = Hardy–Weinberg equilibrium.

Of the 12 haplotypes observed (H1–H12), H1 comprising wild-type alleles at all seven polymorphic sites was the most common, with a significantly higher frequency (Fisher Exact test; *p* = 0.001) in the IMU group (40%) compared to the IMD group (10%) (Table 6). H2, with the allele mutated at position −308, was present in both groups with a lower frequency than H1, but also higher in the IMU group (8%) than in the IMD group (4%) (Table 6). H4, H7, and H8, with alleles mutated at positions −113, −863, and −1031 had a higher frequency in the IMD group (5%, 5%, and 4%, respectively) than in the IMU group (2%, 2%, and 1%, respectively) (Table 6). H3 and H5, which respectively had two mutated alleles (positions −1031 and −863) and one mutated allele (+87), were higher in the IMU group (Table 6). The rare haplotypes: H6, H9, H10, H11, and H12 occurred at a frequency of 1%. Among these, H6, H9, and H10 were only observed in the IMU group, whereas H11 and H12 appeared exclusively in the IMD group (Table 6). However, there was no observed statistically significant difference between the IMD and IMU groups for the H2 –H12 haplotypes.

**Table 6:** Haplotypes (H1 to H12) observed and their frequency according to IMD and IMU groups in patients infected with *S. haematobium*. Haplotype H1 has only wild-type alleles at the segregation sites, but the other haplotypes (H2 to H12) have at least one mutant allele at one of the segregation sites (see highlighted in black).

Haplotype *TNF-α* (dbSNP ID number)	−1031 T > C (rs1799964)	−863 C > A (rs1800630)	−857 C > T (rs1799724)	−308 G > A (rs1800629)	−113 C > G (rs3093660)	+87 G > T (rs2228088)	+489 G > A (rs1800610)	N	IMD	IMU	Fisher test *p*-value
									Frequency (N)	Frequency (N)	
H1	T	C	C	G	C	G	G	44	10% (8)	43% (36)	2.509e–09
H2	T	C	C	**A**	C	G	G	10	4% (3)	8% (7)	0.18
H3	**C**	**A**	C	G	C	G	G	4	1% (1)	4% (3)	0.49
H4	T	C	C	G	**G**	G	G	6	5% (4)	2% (2)	0.57
H5	T	C	C	G	C	**T**	G	4	1% (1)	4% (3)	0.49
H6	T	**C**	**T**	G	C	G	**A**	1	0% (0)	1% (1)	NA
H7	T	**A**	C	G	C	G	G	6	5% (4)	2% (2)	0.57
H8	**C**	C	C	G	C	G	G	4	4% (3)	1% (1)	0.49
H9	T	C	**T**	G	C	G	G	2	0% (0)	2% (2)	0.33
H10	T	C	C	G	C	G	**A**	1	0% (0)	1% (1)	NA
H11	**C**	C	C	G	C	**T**	G	1	1% (1)	0% (0)	NA
H12	T	**A**	C	**A**	C	G	G	1	1% (1)	0% (0)	NA

*N* = sequence number; IMU = infected with morbidity undetected on ultrasound; IMD = infected with morbidity detected on ultrasound. NA = not applicable.

## Discussion

Our results showed an overall average *S. haematobium* prevalence of 24.5%, with variations between villages examined. This prevalence is comparable with that observed in other endemic countries, such as Mali [9], Mauritania, and Senegal [1]. However, this prevalence exceeds the national prevalence observed five years earlier, which was 17.6% [69]. This difference may be attributed to the sampling target, as our study involved larger populations, including adults and out-of-school children, not included in current treatment strategies, showing that these non-targeted groups are actively involved in schistosomiasis transmission and harbor high rates of infection. This further emphasizes the need to extend routing preventive chemotherapy treatment to the entire population in endemic areas, such as the communes of Sô-Ava and Dangbo, in order to achieve the schistosomiasis elimination targets set by the WHO [96].

The morbidity parameters associated with *S. haematobium* infection were validated by both biomarkers and ultrasound analysis. The significant elevation of both ECP and TNF-α concentrations in the urine and plasma of *Sh*+, respectively supports the idea that *Sh* infection induces both systemic and local immune responses. These findings are consistent with previous studies, including those by Masamba and Kappo [53], Asuming-Brempong *et al.* [5], and Wamachi *et al.* [92], who reported activation of inflammatory response during *Schistosoma* infections. The significant correlation between parasite intensity and levels of these immune proteins suggests that they could be used as indicators of parasite load in epidemiological monitoring [61, 78]. Considering urinary disorders measured by ultrasound, over 36% of *Sh*+ showed bladder abnormalities, including bladder wall thickening, frequently observed in chronic *Sh* infections [24, 26, 37, 40, 48]. In contrast, no liver disease was detected, consistent with previous studies showing that periportal fibrosis is specific to intestinal schistosomiasis [54]. Our data differ from those reported by Agniwo *et al.* [3], who observed a high prevalence (56–90%) of bladder irregularities in children in Mali. This could indicate that the disease course is age-related, with bladder thickening observed in adults and more frequent irregularities in children. This mechanism is thought to result from host immune response to eggs trapped in the urinary tract, triggering a Th2 response and the formation of granulation tissue around trapped eggs [14, 18, 87, 103]. Consequently, adults may experience regular egg deposition over the years due to repeated reinfections, leading to the continuous formation of granulation and scar tissue in the bladder, which will ultimately result in thickening of the bladder wall. In contrast, bladder disease in children remains confined to changes in bladder shape, which explains the frequent irregularity observed in this group. Our results also show that infected patients with morbidity detected (IMD) by ultrasound have significantly higher ECP levels than infected patients without morbidity detection (IMU). This phenomenon could indicate more marked activation of immune response in patients with severe forms of the disease. However, plasma TNF-α levels showed no significant difference between IMD and IMU groups, which is similar to the observations of Wamachi *et al.* [92]. The low prevalence of severe disease, such as dilatation of the ureters (3 patients) or kidneys (4 patients), and masses and polyps (2 patients), could suggest that the infection is in a less advanced phase, or that acquired immunity limits the progression of lesions. This could explain the low prevalence of these serious diseases, even though biomarkers indicate immune activation. The observed bladder disorder in *Sh*− participants could also be associated with early treatment, while pathology reversal is still in process, which in most cases takes longer (3 to 6 months in school-age children [10] and 1 to 2 years in adults [49]) than the elimination of urinary egg excretion.

Our findings also highlight a significant association between morbidity status and both the parasite burden and eosinophil-mediated immune activity, but not with systemic TNF-α concentrations. Specifically, individuals classified with mild and moderate morbidity exhibited significantly higher egg counts and elevated ECP levels compared to those in the normal, severe, and very severe groups. This unexpected pattern, where moderate morbidity is associated with higher egg excretion than severe morbidity, may reflect a phenomenon well described in chronic schistosomiasis where the fibrous lesions progressively trap the eggs in the tissues, reducing their excretion in the urine despite the persistence of disease [15, 17, 68, 82]. Similar cases have been observed in patients with *S. mansoni* intestinal schistosomiasis, with advanced liver damage due to chronic inflammation and periportal fibrosis [31]. These findings underscore the limited sensitivity of egg detection methods in chronic stages and the need for complementary biomarkers. The observation of higher ECP levels in mild and moderate groups compared to severe groups points to the key role that active eosinophilic inflammation plays in early or transitional disease [76, 77]. These results are in contrast to the findings of Reimert *et al.* [78] and Leutscher *et al.* [46], who reported a significant increase in urinary ECP levels with progression from mild to severe schistosomiasis morbidity in patients older than 5 years, whereas higher ECP concentrations were observed in individuals between 6 and 19 years than those over 20 years of age [78]. ECP level decline in severe morbidity groups may reflect a shift from inflammatory to fibrotic responses, possibly regulated by immunosuppressive mechanisms such as regulatory T cells or altered cytokine signaling [33, 39, 100]. In contrast, no significant variation in TNF-α levels was observed between morbidity categories. TNF-α plays a central role in granuloma formation and fibrosis in experimental models of schistosomiasis [16]. However, systemic measures may not adequately reflect localized immune responses or may be masked by host immune modulation mechanisms [47], co-infections [72], or chronic immune exhaustion in endemic populations [101]. These results are consistent with previous reports indicating that TNF-α is not a strong systemic marker of disease severity in chronic schistosomiasis [66, 93].

Analysis of *ECP* and *TNF-α* polymorphisms revealed interesting associations between certain genotypes and the parasite egg counts or disease severity. Concerning *ECP*, the different genotypes of the rs2073342 marker were not associated with disease severity. However, patients carrying the rs2073342 GG genotype exhibited significantly higher egg counts in their urine compared to those with other genotypes. Considering that ECP is known to be a marker of eosinophil activation in parasitic infections [4, 84], this suggests that the GG genotype might play a role in modulating parasite intensity, potentially by influencing eosinophil activity. These results are consistent with previous studies that have highlighted the involvement of ECP in immune response, particularly in helminth infections where eosinophils play a critical role in controlling parasite burdens [12, 50, 62, 77].

Genetic variations in the *TNF-α* promoter gene are increasingly recognized as a key factor influencing host susceptibility to severe helminthic infections. In this study, we examined seven polymorphisms previously described in the literature. The absence of significant linkage disequilibrium between markers rs1800629, rs3093660, and rs2228088 located at positions −308, −113, and +87, respectively and the other SNPs analyzed suggests that these variants are not co-segregating. This result confirms the hypothesis that each of these polymorphisms can independently influence host immune response to *Sh* infection. Of these, the G allele at position −113 (rs3093660) was significantly associated with increased morbidity. Individuals homozygous for the G allele (GG genotype) were five times more likely to develop serious clinical manifestations than individuals homozygous for the C allele (CC genotype). This observation is consistent with previous reports suggesting that *TNF-α* promoter polymorphisms can influence TNF-*α* expression, promoting excessive inflammation and tissue damage [89]. In particular, elevated TNF-α levels have been linked to liver fibrosis in schistosomiasis [16, 33]. Other promoter variants such as rs1800629 (−308G > A) and rs361525 (−238G > A) have been shown to alter *TNF-α* transcription and overproduction [99]. Although rs3093660 has been less explored, this study reinforces its potential role in driving granulomatous inflammation and severe immunopathology during helminthic infections. Surprisingly, this study found no significant difference in plasma TNF-α levels between patients carrying the CC and GG genotypes of the rs3093660 polymorphism (Supplementary file 2). However, ECP levels were significantly higher in patients carrying the GG genotype than in those carrying the CC genotype. These results suggest that this variant of the *TNF-α* gene promoter may modulate ECP expression by indirect mechanisms. This observation raises an important question: how could the G variant of the rs3093660 gene influence ECP levels without altering systemic TNF-α concentrations? Several plausible mechanisms can be envisaged: (i) local modulation: the G allele of rs3093660, located in the *TNF-α* promoter region, may selectively affect local cytokine expression and eosinophil activation in inflamed tissues [27, 67]; (ii) cytokine synergy: the variant can indirectly modulate eosinophil responses by interacting with the IL-4 and IL-5 signaling pathways, which are known to lead to the release of ECP [28, 63]; and (iii) post-transcriptional regulation: *TNF-α* is subject to complex regulatory mechanisms, including mRNA stability, translation efficiency, and protein turnover, which can dissociate the effects of promoter polymorphism from plasma cytokine levels [23, 36, 41, 52].

In terms of haplotype analysis, in the study, we identified 12 haplotypes based on the seven mutations in the *TNF-α* gene. The H1 haplotype, carrying wild-type alleles at all seven mutation sites, was the most common and showed a significantly higher frequency in the IMU group compared to the IMD group. This suggests that the H1 haplotype may be protective or associated with less severe disease. In contrast, haplotypes H4, H7, and H8, which carried mutations at positions −113, −863, and −1031, were more frequent in the IMD group, suggesting that these haplotypes may be linked to morbidity. Our results highlight the complex influence of host genetic variation on immune regulation and progression of schistosomiasis, ultimately shaping patterns of morbidity and clinical severity.

Although this study provides valuable information on the genetic and immunological mechanisms underlying schistosomiasis, it has several limitations that should be taken into account in future research. First, the ultrasound assessments were carried out on only 146 patients, which may limit the generalizability of the results. To address this, future studies should aim to include larger and more diverse populations in different endemic regions, allowing for more robust and representative assessments of morbidity patterns. Second, TNF-α levels were not measured directly at sites of infection (*e.g.*, in urine samples), limiting our ability to assess local cytokine activity. Incorporating tissue-specific or excretory biomarker measurements into future research would provide clearer data on local immune responses and inflammation. Third, the overall sample size included in the genetic association analysis was relatively small, potentially limiting the statistical power to detect subtle genetic associations. Increasing the size of the cohort through multicenter collaborations would increase analytical power and improve detection of genotype-phenotype correlations. Although our genetic analysis focused on known polymorphisms, it might have missed rare or novel variants. Therefore, the use of high-throughput genomic approaches, such as next-generation sequencing (NGS), could enable more comprehensive exploration of the genetic loci underlying disease severity. Fourth, the cross-sectional nature of the study does not allow us to understand the temporal dynamics of infection or immune responses. Longitudinal cohort studies that monitor changes in biomarkers and morbidity over time would help to clarify causal relationships and the progression of disease.

In conclusion, this study highlights the complex interaction between *Sh* infection, immune response, and genetic factors. Biomarkers such as ECP and TNF-α could potentially be used as reliable indicators to assess disease severity. Furthermore, genetic polymorphisms, in particular the rs3093660 variant of the *TNF-α* gene, are emerging as relevant predictive markers for identifying patients at risk of severe forms, enabling clinicians to adapt therapeutic strategies. Finally, the study highlights the determinant role of genetic factors in susceptibility to urinary schistosomiasis and its clinical course. Further research is still needed to validate these genetic associations and clarify their impact on disease progression.

**Abbreviations**
*Sh*
: *Schistosoma haematobium*
*Sh*+: Patients infected with *Sh*
*Sh*−: Patients uninfected with *Sh*
*Sm*
: *Schistosoma mansoni*
IL: interleukin
DNA: Deoxyribonucleic acid
SNP: Single nucleotide polymorphism
TNF-α: Tumor necrosis factor-alpha
ECP: Eosinophil cationic protein
CNERS: Comité national d’éthique pour la recherche en santé
IMU: Infected with morbidity undetected on ultrasound
IMD: Infected with morbidity detected on ultrasound
*p*
: *p*-value
LD: linkage disequilibrium
RFLP-PCR: Restriction fragment length polymorphism - polymerase chain reaction
U: Mann–Whitney statistic test
KW: Kruskal–Wallis statistic test
GLM: Generalized linear models
χ^2^: Chi-Square test

## Figures and Tables

**Supplementary file 1:**
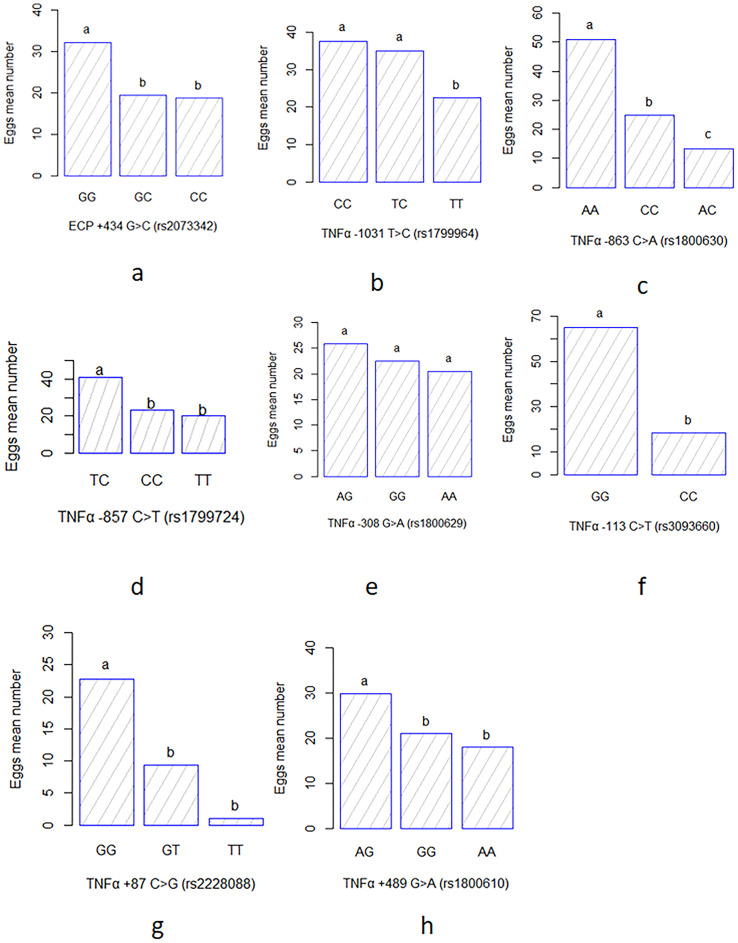
SNP marker genotype structuring analysis based on average number of eggs in patients infected with *S. haematobium.*

**Supplementary file 2:**
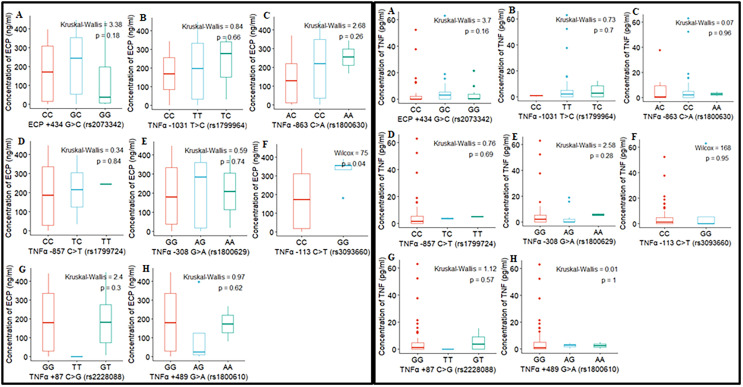
Comparative analysis of urinary ECP (left) and plasma TNF-α concentrations (right) according to the genotypes identified for each marker in samples from patients infected with *S. haematobium.*

## Data Availability

All data generated or analyzed during this study are included in this published article.

## Supplementary material

Analysis of the structure of the SNP marker genotypes was carried out according to the average number of eggs obtained after the GLM analysis (see Table 4). On the histograms of the genotypes, the presence of identical letters (“a” or “b”) above the bars indicates that there is no significant difference between these genotypes (e). On the other hand, different letters indicate a statistically significant difference (f).

The supplementary material of this article is available at https://www.parasite-journal.org/10.1051/parasite/2026013/olm.
